# Effects of exercise interventions on sleep quality in adolescents: a systematic review and meta-analysis

**DOI:** 10.3389/fpubh.2025.1623506

**Published:** 2025-09-03

**Authors:** Haoming Yan, Guangjie Xin, Rui Chen

**Affiliations:** ^1^School of Physical Education, Chengdu Sport University, Chengdu, China; ^2^College of Physical Education and Health Sciences, Zhejiang Normal University, Jinhua, China; ^3^School of Physical Education and Sport Science, Fujian Normal University, Fuzhou, China

**Keywords:** exercise interventions, adolescent, sleep quality, meta-analysis, subgroup analysis

## Abstract

**Objective:**

Adolescents’ declining sleep quality has caught the attention of educators, parents, doctors, and schools. Research, especially focusing on teenagers, is still lacking, despite numerous studies examining the connection between physical exercise and sleep quality across other demographics. This study aims to systematically review and conduct a meta-analysis to evaluate the impact of exercise interventions on adolescents’ sleep quality.

**Methods:**

Investigate articles available until April 17, 2025, in databases such as PubMed, Cochrane, Web of Science, EBSCO, and Embase. Evaluate the quality of the studies based on the standards outlined in the Cochrane Handbook and conduct data analysis with Review Manager 5.3 software.

**Results:**

After screening 2,312 articles, 8 studies involving 710 participants were ultimately included for meta-analysis. The meta-analysis results indicated that exercise effectively improves sleep quality in adolescents (SMD = −2.10, 95% CI: −2.86 to −1.35, *p* < 0.05). Various weekly exercise frequencies, single-session lengths, and total intervention durations all demonstrated statistically significant favorable impacts on adolescents’ sleep quality, according to subgroup analyses. Regarding exercise modality, combined exercise demonstrated no significant effect on adolescents’ sleep quality, whereas both aerobic and resistance exercise produced significant improvements.

**Conclusion:**

Exercise can effectively improve sleep quality in adolescents. A systematic exercise program lasting 3 to 12 weeks is beneficial for the sleep quality of adolescents, with the maximum benefit achieved at 12 weeks. Conducted at an appropriate frequency and with each session lasting more than 30 min, it is particularly effective in improving the sleep quality of adolescents. Both aerobic and resistance exercise significantly improve adolescents’ sleep quality, whereas combined exercise shows no significant effect. Parents and educators should see exercise treatments as a practical, secure, and efficient non-pharmacological way to improve adolescents’ sleep quality.

**Systematic review registration:**

https://www.crd.york.ac.uk/PROSPERO/view/CRD420251033597, Identifier CRD420251033597.

## Introduction

Sleep is a fundamental biological process that underpins healthy growth, neurocognitive development, and emotional regulation during adolescence (1). High-quality sleep is essential for adolescents’ health, learning, memory, and vitality, and it also exerts a profound influence on their overall well-being (2, 3). Regrettably, an increasing proportion of adolescents fail to meet the recommended sleep-duration guidelines (4), and more than 70% experience insufficient sleep on school days (5). Adolescents who get too little or poor quality sleep are more likely to have mental problems, metabolic dysregulation, increased risk-taking behavior, and poor academic performance, among other negative consequences (6–8). Furthermore, poor sleep quality may lead to weakened immunity, impaired nervous system function, elevated obesity rates, and higher disease incidence among adolescents (9). Prolonged sleep deprivation can also exacerbate negative emotions such as anxiety, depression, and irritability, thereby severely undermining mental health (10). Accordingly, improving adolescent sleep quality has become an urgent priority for clinicians and educators.

In clinical practice, common strategies to improve sleep include pharmacological and non-pharmacological treatments. Common drugs that have been shown to improve sleep include melatonin, antipsychotics, calcium antagonists, and antidepressants (11, 12). However, prolonged usage of these medications may result in negative consequences, including tolerance, dependency, and stigma, all of which can have a significant negative influence on one’s quality of life (13, 14). Therefore, non-pharmacological therapies may offer more enduring efficacy and lower life risks compared to drug treatments, making them more advisable for adolescents with insufficient sleep quality (15).

Most people agree that exercise is essential for fostering long-term health. The changes it induces in immune function, nervous system recovery, body temperature regulation, endocrine function, metabolic processes, and circadian rhythms collectively contribute to the initiation and maintenance of sleep (16–18). Furthermore, exercise therapy is generally regarded as a low-cost, safe, and promising measure for improving sleep (19). A comprehensive analysis suggests that different types of exercise are commonly utilized by people experiencing sleep issues and may, to a certain degree, act as a substitute for pharmaceuticals in enhancing sleep quality and increasing sleep duration (20). Other research, however, has drawn attention to the paucity of reliable data on how healthy exercise might enhance adolescent sleep (21). These conflicting findings present significant challenges to clinical practices aimed at enhancing sleep quality among adolescents.

Previous narrative reviews have extensively summarized the relationship between exercise therapy and sleep, indicating that exercise is beneficial for individual sleep (22, 23). However, to our knowledge, current research exploring the relationship between exercise and sleep primarily focuses on adults and the old population, and there is a lack of comprehensive systematic reviews and meta-analyses specifically targeting exercise interventions for adolescent sleep outcomes. Such evaluations are crucial not only for elucidating the magnitude and clinical relevance of sleep improvements induced by exercise but also for identifying optimal intervention characteristics and evidence gaps that should guide future research. This research aims to assess the effects of exercise therapy on sleep among adolescents by conducting a systematic review and meta-analysis. This evaluation seeks to guide clinical practices, inform public health recommendations, and aid in planning future intervention studies that promote healthy sleep in this age group.

## Methods

### Registration

To ensure methodological rigor and preserve scientific integrity, this meta-analysis meticulously adhered to the principles specified in the Preferred Reporting Items for Systematic Reviews and Meta-Analyses (PRISMA) statement (24) during the analysis and the report’s writing. The protocol for this review has been registered with the PROSPERO platform, identified by registration number CRD420251033597.

### Search strategy

A thorough examination of pertinent literature was carried out by searching through five databases (namely PubMed, Embase, Cochrane Library, EBSCO, and Web of Science) utilizing computer-assisted retrieval, with the search covering data until April 17, 2025. To improve the search’s thoroughness, we used four groups of keywords: (1) exercise, physical activity, physical training, aerobic exercise, physical exercise, Baduanjin, Qigong, Tai Chi, Yoga, Pilate, resistance training, physical training, strength training, walk, swim, fitness; (2) sleep quality, sleep, sleep maintenance, sleep disorder, sleep problem, insomnia, sleeplessness, sleep duration, sleep health; (3) adolescent, youth, teen, school student; (4) randomized controlled trial, randomized, placebo. These keywords were connected using Boolean logical operators. We also looked through the reference lists of the journals we collected to locate relevant research. At the same time, we performed citation tracking by looking at the studies featured in existing systematic reviews and meta-analyses, as well as their reference lists, to guarantee that our search was thorough. Comprehensive search strategies are available in the Supplementary material.

### Inclusion and exclusion criteria

This study established rigorous criteria for the inclusion and exclusion of literature, ensuring that all articles selected during the screening process adhered to the PICOS principle. The inclusion criteria for the literature are as follows: (1) Population: Individuals aged between 10 and 19 years. (2) Intervention: The experimental group engaged in a structured exercise program that incorporated aerobic, resistance, and comprehensive exercises, allowing for various modes, intensities, durations, and frequencies. (3) Comparisons: The control group received normal treatment, health education, or their regular daily routines instead of engaging in any exercise interventions. (4) Outcomes: Various standardized measures, including the Pittsburgh Sleep Quality Index (PSQI), Epworth Sleepiness Scale (ESS), and Insomnia Severity Index (ISI), alongside objective assessment tools such as sleep electroencephalography (EEG), were utilized to evaluate pertinent indicators of sleep quality. (5) Study design: Only randomized controlled trials (RCTs) were included to gather high-level data.

The evaluation procedure was conducted using the following criteria for exclusion: (1) Trials that were not randomized and controlled. (2) Studies that did not include a control group. (3) Experiments involving animals. (4) Outcomes related to sleep that were not measured. (5) Involvement of a control group not being present. (6) Conference abstracts, observational research, theses, and correspondence. (7) Lack of data that could be extracted.

### Data extraction

This research was conducted independently by two researchers (HM-Y and RC). All bibliographic records were transferred into EndNote X9 from various databases, and any duplicate entries were systematically eliminated. An initial assessment focusing on the titles was then conducted to filter out literature that was evidently not related to the research topic. After this, a thorough evaluation was executed following predefined screening criteria to identify studies that aligned with the eligibility requirements. In situations where there was a disagreement during the screening phase, a third researcher (GJ-X) was invited to contribute to the discussion, with data extraction taking place only once an agreement was achieved among all three individuals. The collected indicators cover several key dimensions, specifically including: details of the first author, publication date of the literature, sample size, ages of both experimental and control groups, details of the intervention methods, duration of the intervention, frequency, intensity parameters, and measurement data related to sleep indicators.

### Risk of bias assessment

Two researchers independently conducted the quality assessment. If any inconsistencies arose, a third researcher would be consulted for further discussion to arrive at a conclusive decision. Assess each trial’s methodological quality using the Cochrane Collaboration’s suggested bias risk criteria (25). This evaluation tool examines multiple aspects, such as the randomness of the allocation sequence, concealment of the allocation scheme, blinding of participants and researchers, blinding in outcome assessment, completeness of outcome data, selective outcome reporting, and any other relevant biases. There were three categories for each risk of bias criterion: low, high, and unclear.

### Statistical analysis

The analysis of continuous variable data was conducted using Review Manager 5.3 software (26). Based on the specific measurement techniques and units of the variables, the effect measure chosen was either the standard mean difference (SMD) or the weighted mean difference (WMD), accompanied by the 95% confidence interval (CI) for the statistical evaluation (27). The I^2^ statistic was utilized to evaluate the heterogeneity among the studies. When heterogeneity was minimal or absent (*p* > 0.1, *I*^2^ < 50%), a random-effects model was utilized for the analysis; in contrast, a fixed-effects model was employed in cases of greater heterogeneity (28). The leave-one-out strategy was used for sensitivity analysis in meta-analyses with four or more studies (29). It was determined that the elimination of a single study had a significant influence if it changed the pooled findings in a meaningful way. Statistical significance was defined as a *p*-value of less than 0.05.

## Results

### Literature search results

**Figure 1:**
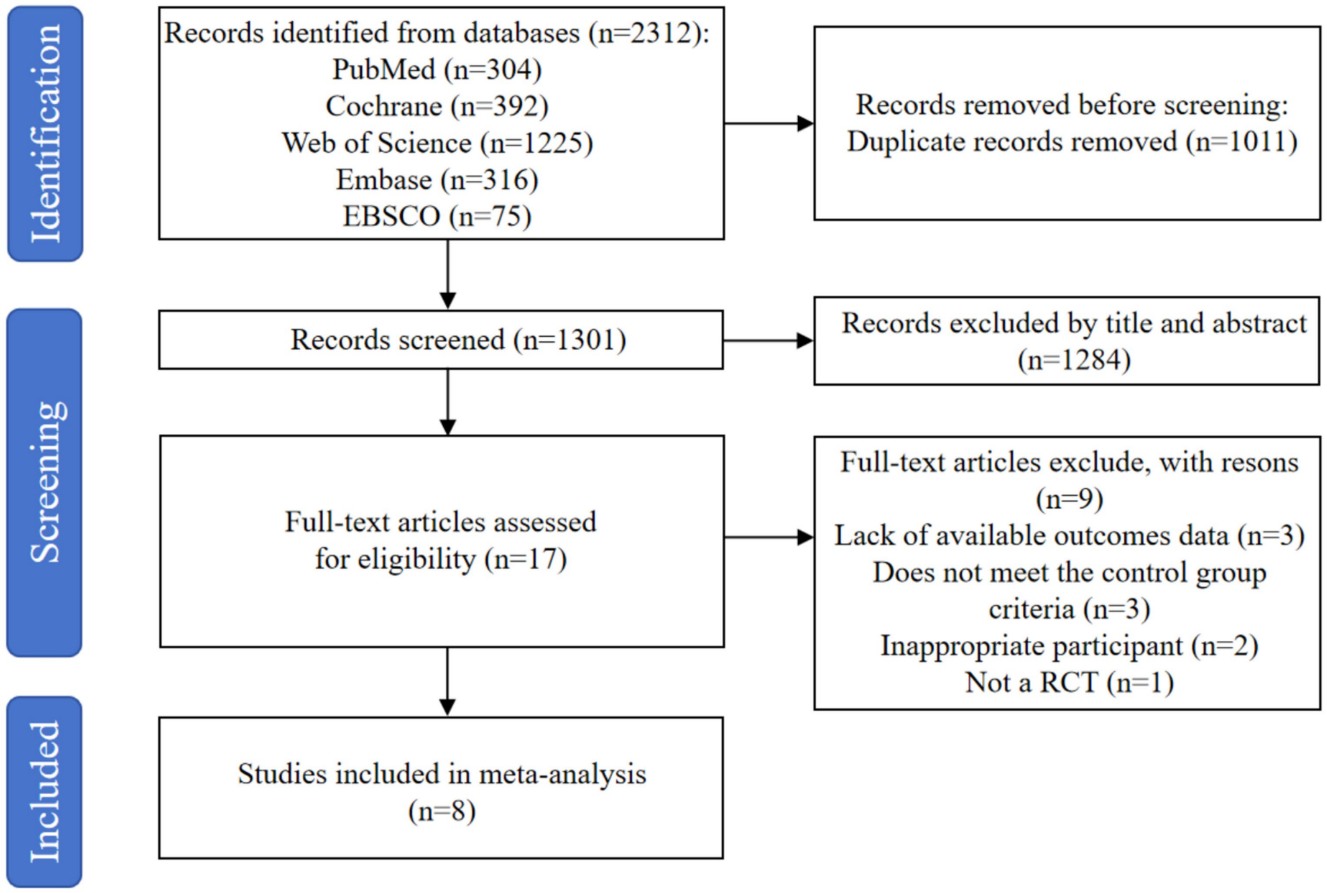
Flow diagram of literature selection process.

The research involved searching through five databases, which yielded a total of 2,312 records. After eliminating 1,011 duplicate entries with the help of the EndNote X9 reference management software, 1,301 records were left for further consideration. After a preliminary screening based on the abstracts and titles, 1,284 judged unnecessary records were eliminated. A comprehensive review of the full texts of the remaining 17 records took place, resulting in the discarding of 9 articles for the following reasons: lack of available outcomes data (*n* = 3), does not meet the control group criteria (*n* = 3), inappropriate participant (*n* = 2), and not a RCT (*n* = 1). In the end, 8 studies (30–37) were incorporated into the meta-analysis. Figure 1 shows the literature screening procedure.

### Basic characteristics of the included literature

The study’s primary characteristics are illustrated in Table 1. All studies were conducted in regions such as Switzerland, Iceland, Brazil, China, India, and Taiwan, China, and were published between 2012 and 2025. The participants in these studies were adolescents aged 10–19 years (38). The exercise interventions for the experimental groups included activities such as running, walking, strength training, Pilates, integrated training, and sports games. The duration of the intervention programs ranged from 3 to 12 weeks. Each exercise session occurred with frequencies ranging from twice weekly to seven times weekly, with three sessions per week being the most common. Session durations varied from 3 to 75 min; however, one study did not specify intervention times as it focused on recording participants’ daily walking activities (31 (Table 1).

**Table 1:** Characteristics of eligible studies.

Included studies	Country	Research classification	Sample size (N)	Age of participants	Intervention		Intensity	Dose	Outcome measures
					E	C			
Kalak et al. (2012) (30)	Switzerland	RCT	EG = 27; CG = 24	18.30 ± 0.89	Running	Normal lifestyle	Moderate	3 weeks; 30-min/session; 7 times/week	Sleep-EEG, the Insomnia Severity Index, Subjective sleep quality
Baldursdottir et al. (2017) (31)	Iceland	RCT	EG = 26; CG = 27	15–16	Walking	Normal lifestyle	Moderate	3 weeks; 7 times/week	Subjective sleep quality
Santiago et al. (2020) (32)	Brazil	RCT	EG = 18; CG = 12	16.0 ± 1.4, 16.4 ± 1.6	Strength training	Non-exercise	NR	12 weeks; 55-min/session; 3 times/week	PSQI, ESS
Hu et al. (2023) (33)	China	RCT	EG = 26; CG = 26	16–19	Aerobic exercise	Normal lifestyle	Moderate	12 weeks; 75-min/session; 4 times/week	PSQI
Parveen et al. (2024) (34)	India	RCT	EG = 30; CG = 30	15.23 ± 2.48	Pilates	Normal lifestyle	Low to moderate	8 weeks; 60-min/session; 3 times/week	PSQI
Wang et al. (2024) (35)	China	RCT	EG = 45; CG = 45	10.95 ± 0.43, 10.98 ± 0.62	Aerobic calisthenics and resistance exercises	Non-exercise	Moderate	8 weeks; 40-min/session; 3 times/week	PSQI
da Silva et al. (2025) (36)	Brazil	RCT	EG = 165; CG = 141	13.5 ± 0.96	Aerobic and resistance exercises	Routine activities	NR	12 weeks; 20-min/session; 2 times/week	PSQI
Kao et al. (2025) (37)	Taiwan, China	RCT	EG = 35; CG = 33	10.76 ± 0.49	Sports games	Routine activities	Moderate- to vigorous	8 weeks; 3-min/session; 3 times/week	PSQI

### Quality assessment

**Figure 2:**
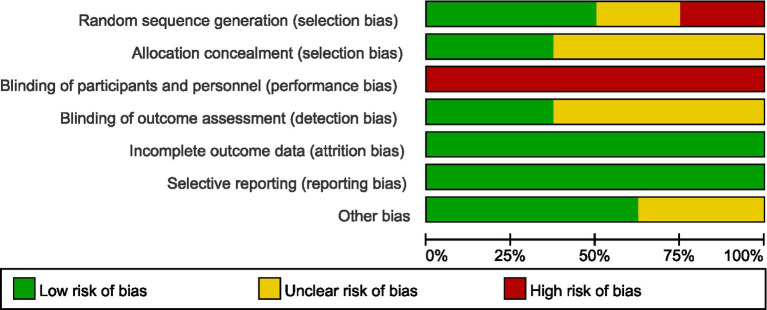
Overall overview graph of bias risk in included studies.

**Figure 3:**
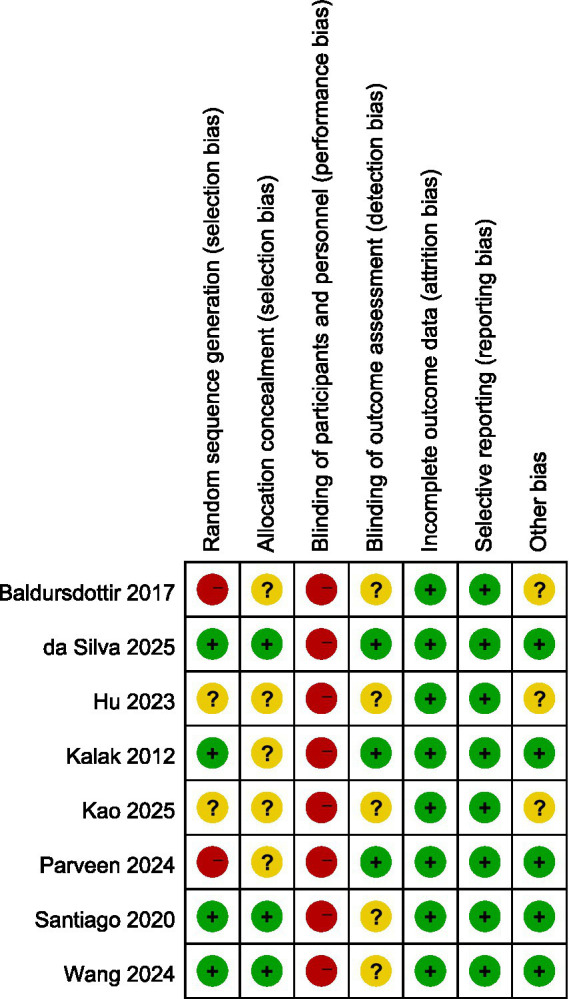
Risk of bias evaluation graph for the included literature.

The evaluation of bias risk employed the tool recommended by the Cochrane Collaboration, with results for the studies included illustrated in Figures 2, 3, respectively. Out of the 12 studies analyzed, two were found to possess a high bias risk related to the method of random allocation. The inability to blind participants regarding the exercise intervention (comparing exercise to no exercise) resulted in a high risk of bias from both participants and personnel across all research. The remaining aspects did not reveal any high risk of bias. All studies showed strong performance concerning the completeness of outcome data and the reporting of anticipated outcome measures. In conclusion, the quality evaluation found that every study that was included had a low to moderate risk of bias. The duration of the intervention programs ranged from 3 to 12 weeks.

### Result of meta-analysis

#### Overall sleep quality

**Figure 4:**
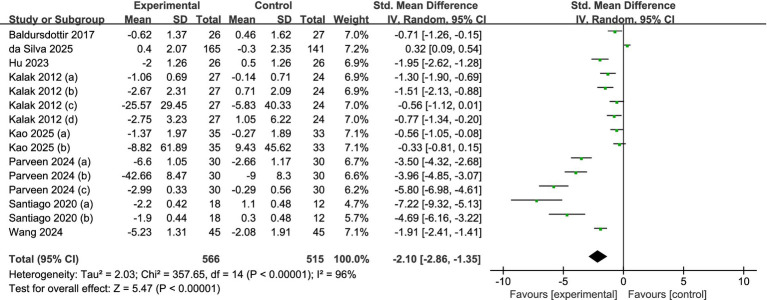
Forest plot of the overall effect of exercise intervention on sleep quality in adolescents.

A meta-analysis of the entire sample across all reviewed studies suggests that exercise significantly enhances the sleep quality among adolescents. Figure 4 illustrates that the findings from the meta-analysis suggest a significant enhancement in adolescents’ sleep quality due to exercise. The calculation of the overall effect size using a random-effects model produced a heterogeneity test result with an *I*^2^ = 96% (*p* < 0.01). This substantial degree of heterogeneity indicates that various moderating factors might affect the overall impact observed in the studies examined. Figure 4 illustrates that the overall standardized mean difference (SMD = −2.10, 95% CI = −2.86 to −1.35, *p* < 0.001) suggests a significantly greater enhancement in sleep quality for the exercise group compared to the control group.

### Subgroup analysis

#### Subgroup analysis of intervention frequency

**Figure 5:**
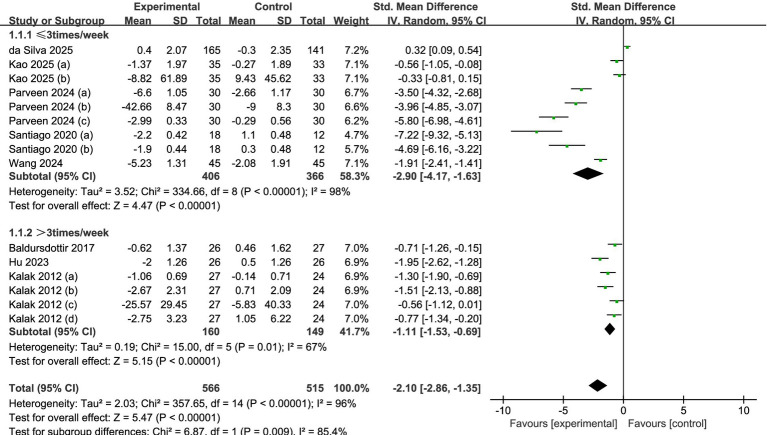
Forest plot of subgroup analysis by intervention frequency.

A total of eight studies were analyzed (see Figure 5). Among these, five studies (32, 34–37) reported data collected at a frequency of three times per week or fewer, while three studies (30, 31, 33) reported data at a frequency exceeding three times per week. The results of the analysis, employing a random-effects model, revealed that, irrespective of whether the frequency was less than three times per week (SMD = −2.90, 95% CI = −4.17 to −1.63, *p* < 0.001) or more than three times per week (SMD = −1.11, 95% CI = −1.53 to −0.69, *p* < 0.001), significant statistical differences between the experimental and control groups suggested that the adolescents in the experimental group experienced enhanced sleep quality.

#### Subgroup analysis of each intervention time

**Figure 6:**
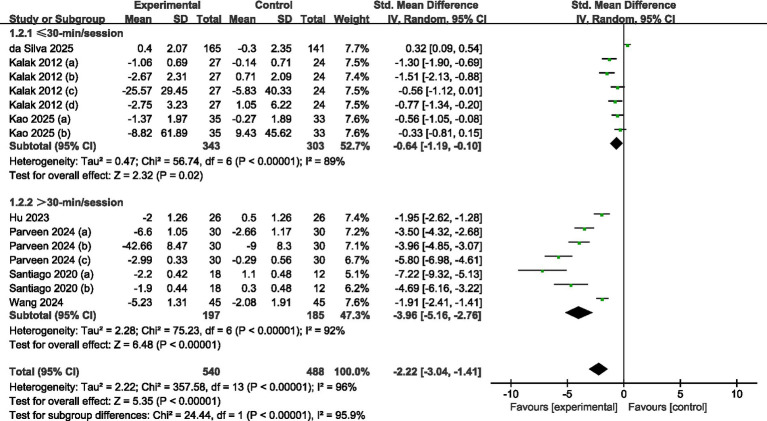
Forest plot of subgroup analysis by each intervention time.

A total of seven studies were reviewed (refer to Figure 6). Among these, three studies (30, 36, 37) provided data regarding sessions shorter than 30 min, whereas four studies (32–35) shared data on sessions that lasted longer than 30 min. Using a random effects model, the analysis revealed significant statistical differences between the experimental and control groups for both the longer sessions (SMD = -3.96, 95% CI = −5.16 to −2.76, *p* = 0.001) and the shorter sessions (SMD = -0.64, 95% CI = −1.19 to −0.10, *p* = 0.02). This suggests that the intervention significantly impacted the enhancement of sleep quality (*p* < 0.001). Regarding effect size, sessions extending beyond 30 min proved to be more effective in improving sleep quality among adolescents.

#### Subgroup analysis of the total intervention duration

**Figure 7:**
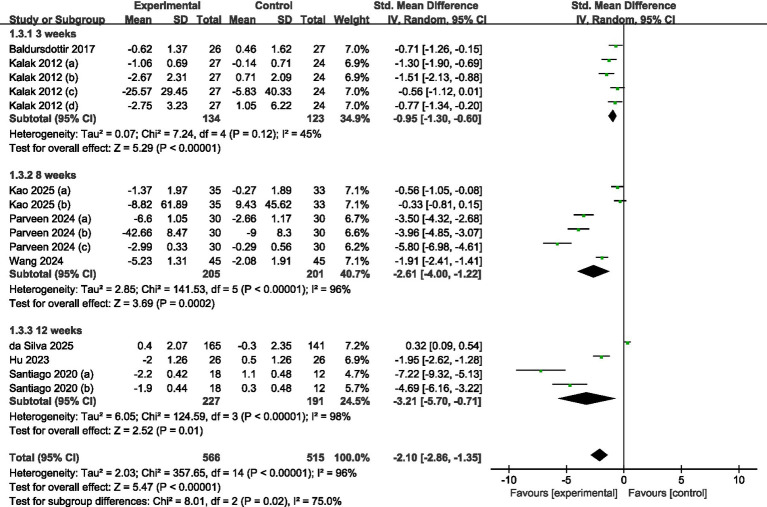
Forest plot of subgroup analysis by total intervention duration.

A total of eight studies were incorporated (refer to Figure 7). Among these, two studies (30, 31) provided data following 3 weeks of intervention, while three studies (34, 35, 37) disclosed findings after 8 weeks, and the final three studies presented data after 12 weeks of intervention (32, 33, 36). The analysis, which utilized a random-effects model, indicated that there were statistically significant differences in sleep quality between the intervention and control groups at 3 weeks (SMD = −0.95, 95% CI = −1.30 to −0.60, *p* < 0.001), 8 weeks (SMD = −2.61, 95% CI = −4.00 to −1.22, *p* < 0.001), and 12 weeks (SMD = −3.21, 95% CI = −5.70 to −0.71, *p* = 0.01). The most substantial effects were observed at 12 weeks.

#### Subgroup analysis of types of exercise intervention

**Figure 8:**
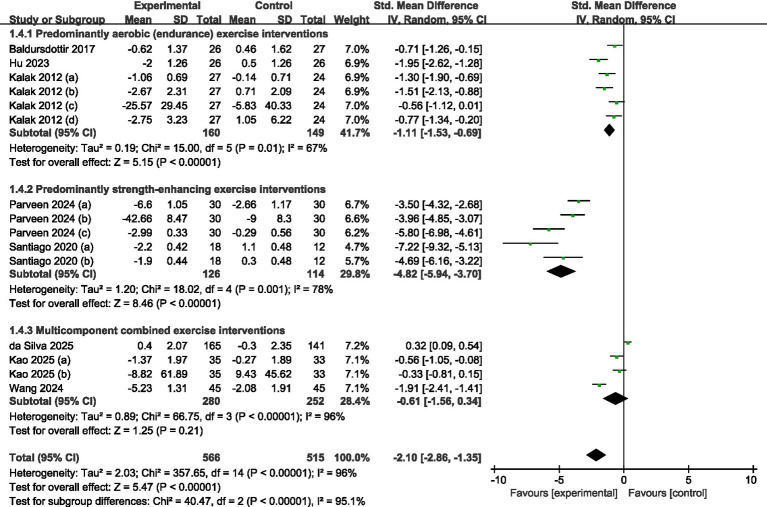
Forest plot of subgroup analysis by exercise type.

Eight studies in all met the requirements for inclusion and were included in the subgroup analysis (refer to Figure 8). Specifically, three trials (30, 31, 33) focused on predominantly aerobic exercise, two (32, 34) centered on predominantly strength-enhancing exercise, and the remaining three (35–37) assessed multicomponent combined exercise programs. Random-effects modeling showed that both aerobic regimens (SMD = −1.11, 95% CI = −1.53 to −0.69, *p* < 0.001) and strength-enhancing regimens (SMD = −4.82, 95% CI = −5.94 to −3.70, *p* < 0.001) produced statistically significant gains in sleep quality among adolescents. Conversely, multicomponent combined exercise did not yield a significant benefit (SMD = −0.61, 95% CI = −1.56 to −0.34, *p* > 0.05).

#### Sensitivity analysis and publication bias

**Figure 9:**
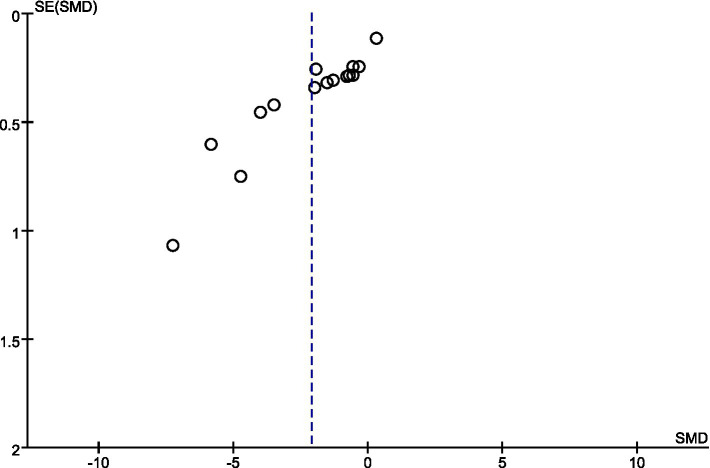
Publication bias analysis results.

An analysis of sensitivity was performed on the sleep indicator data that participants assessed themselves. The findings indicated that excluding any one independent study did not result in statistically significant alterations in the combined effect size and its 95% confidence interval (CI). This result demonstrates the strong dependability of exercise’s impact on measures of adolescent sleep quality. The assessment of potential publication bias in studies with small sample sizes was conducted through the funnel plot method (39). Figure 9 illustrates that the data points are positioned on either side of a downward-facing funnel shape, exhibiting a mostly even distribution but with a small number of dispersed points. This result implies that the degree of publication bias risk is still within an acceptable range, even if it may have existed in the eight papers that were reviewed.

## Discussion

With a focus on randomized controlled trials (RCTs), this systematic review and meta-analysis examined how exercise treatments affected adolescents’ sleep quality. After thorough screening, we identified 8 studies that fulfilled the inclusion criteria from an original pool of 2,312 studies. We extracted outcomes related to sleep quality from these studies to assess variations in sleep quality subsequent to exercise interventions. As far as we are aware, a thorough meta-analysis assessing the effects of exercise on the sleep quality of adolescents has not been conducted. Consequently, this research seeks to furnish more detailed and reliable evidence to summarize and validate the genuine importance of exercise on the sleep patterns of adolescents.

The results from our research show that the group that participated in the exercise intervention experienced a notable enhancement in overall sleep quality when compared to the control group (SMD = −2.10, *p* < 0.001). Subgroup analysis was used to further examine the effects of the intervention; however, because of the substantial heterogeneity caused by the small number of included studies, care should be used when interpreting the subgroup analysis’s findings.

According to the frequency of interventions, this study separated the body of current material into two main categories: “three times a week or fewer” and “more than three times a week.” The results of the analysis suggest that exercise significantly enhances adolescents’ sleep quality, irrespective of the frequency of intervention. This effect may arise from the enduring regulatory influence of exercise on emotional states (40). Affective regulation mechanisms appear to reach an early plateau. Even a relatively short exercise schedule (no more than 3 times a week) is sufficient to trigger sizeable, sustained elevations in endorphins, brain-derived neurotrophic factor, and serotonin, while concurrently dampening hypothalamic–pituitary–adrenal (HPA) axis reactivity (41, 42). These changes in neurochemicals collectively alleviate negative emotions such as anxiety, depression, and irritability, which are the stressors most closely associated with adolescent sleep disorders (43, 44). Once this neuroendocrine “threshold” is crossed, additional weekly treatments have diminishing returns in terms of mood improvement and sleep. Additionally, previous evidence suggests a modest difference between exercising three times a week and exercising more frequently; however, both approaches significantly improve sleep quality (45). However, if the weekly dose is higher, there may be a ceiling or even a reversal effect (46). Adolescents who exercise more often have higher cumulative energy expenditure and core temperature loads; for those who are already under pressure from demanding academic schedules, this extra strain shortens evening routines, postpones sleeping, and partly counteracts the expected sleep benefits.

In the subsequent stage, the study divided the intervention into two groups based on the duration of each intervention: “30 min and below” and “more than 30 min.” Findings revealed that exercise durations exceeding 30 min greatly influenced the improvement of sleep among adolescents. Due to their higher energy levels, adolescents can maintain quality exercise for extended periods. Exercise lasting longer than 30 min effectively boosts dopamine production in their bodies (47). Enhanced dopamine levels help regulate sleep by modifying the circadian rhythms of various biological clock genes, interacting with internal substances like adenosine, and providing neuroprotective benefits through alterations in neuronal structures, which aids in reducing sleep disorders and improving overall sleep quality (48, 49). Furthermore, extended periods of exercise grant adolescents additional time to release negative emotions, which further enhances sleep-related advantages. Additionally, extended periods of exercise may result in a reduction of a person’s core body temperature (50). The regulation of body temperature is essential for sleep, as it assists in initiating sleep signals, minimizing sleep onset time, and enhancing the length of deep sleep (51, 52). Ultimately, participating in over 30 min of exercise can offer adolescents additional opportunities to ease negative feelings, thus creating further advantages that promote better sleep (53).

The duration-based subgroup analysis revealed that adolescents’ sleep quality can improve markedly within a short timeframe. Consistent with this finding, previous work has shown that as little as three consecutive days of structured exercise are sufficient to elicit measurable benefits (54). This early response is physiologically plausible: even a short period of exercise can raise evening melatonin concentrations, up-regulate anti-inflammatory cytokines, and lower pro-inflammatory markers, thereby increasing slow-wave sleep and sleep efficiency (55). Furthermore, consistent and regular exercise might indirectly increase melatonin secretion, boost general well-being, and improve the quality of sleep (56). The eight-week exercise program yielded more outstanding results, with a greater increase in effect size. The 12-week exercise intervention had the most significant positive impact on sleep quality, but the magnitude of the increase in effect size slightly diminished. This progressive attenuation may represent a physiological adaptation: when the training load is kept constant over time, improvements in fitness reduce the relative intensity of each session, thereby diminishing the physiological stimulus that promotes sleep (57, 58). Moreover, longer intervention periods impose higher demands on scheduling (59). For adolescents with heavy academic workloads, exercising late in the day may encroach on their allotted sleep window, provoking delayed sleep onset, reduced total sleep time, and circadian disruption (60). However, overall, a regular, long-term exercise program still exerts the most significant positive impact on adolescent sleep quality, making it the optimal choice for improving sleep in adolescents.

Ultimately, this research revealed that resistance exercise is the most effective form of exercise intervention, with aerobic exercise following closely behind, aligning largely with earlier studies (61). Interestingly, the anticipated effectiveness of combined exercise did not meet expectations, diverging from findings in previous research (62). This discrepancy is related to the characteristics of the studies included, with the lower intervention frequency and shorter duration of each training session in the combined exercise group being significant contributing factors to the lack of pronounced effects. This could be attributed to a lower frequency of intervention and shorter duration for each session. Research in the scientific field has shown that infrequent exercise and brief exercise sessions have minimal effects on enhancing the physical and mental well-being as well as the life satisfaction of young people, which in turn affects the efficacy of enhancing sleep quality (63). There is evidence that aerobic exercise has significant positive impacts on the structure and function of the brain (64). For example, aerobic activity has been linked to an increase in the size of the hippocampus and a rise in the volume of different gray and white matter locations inside the frontal and temporal lobes (65). It can also enhance cerebral blood flow, and upregulate brain-derived neurotrophic factor (BDNF) which is closely associated with learning, memory, and executive function, thereby alleviating daytime sleepiness and insomnia symptoms while improving overall sleep efficiency (66, 67). Moreover, aerobic exercise may lessen the nocturnal rostral fluid shift, strengthen respiratory muscles, and improve upper airway patency, all of which contribute to unobstructed breathing and higher-quality sleep (68–70). Melatonin is a hormone that is directly linked to people’s inclination to sleep, and new research indicates that resistance exercise raises melatonin levels more than aerobic exercise (55). The considerable rise in melatonin resulting from resistance exercise promotes positive alterations in a person’s sleep–wake rhythm, leading to a decrease in the number of nighttime awakenings (71). Additionally, it has been shown to increase serum levels of anti-inflammatory cytokines while more effectively lowering levels of the pro-inflammatory cytokine IL-6 and circulating pro-inflammatory markers in the body, which helps to alleviate certain sleep disturbances (72, 73). In addition, resistance exercise counteracts the muscle atrophy that follows sleep deprivation, slows sarcopenic processes, and enhances overall physical fitness, all of which promote greater myokine release and amplify anti-inflammatory effects (74–76). Furthermore, it has been demonstrated that both aerobic workouts and resistance exercise considerably increase the amounts of anti-inflammatory cytokines, which in turn supports both physical and mental well-being (77, 78). Taken together, these findings indicate that a scientifically balanced combination of aerobic and resistance exercises is theoretically poised to deliver superior improvements in sleep quality.

This review has several limitations. First, the modest sample sizes and limited number of eligible studies produced substantial heterogeneity, which may compromise the precision of our estimates; therefore, care should be used while interpreting the results. Secondly, due to the limited number and characteristics of the trials included, subgroup analysis could only account for a small portion of this heterogeneity. Particularly in terms of exercise modalities, the limitations in the characteristics of the included studies led to some discrepancies with previous research, thereby affecting the generalizability of the conclusions to a certain extent. Third, most trials did not employ double-blind designs, introducing potential bias in outcome assessment. Fourth, language and source bias resulted from our search strategy’s restriction to peer-reviewed English-language publications. Future work should broaden the search to include studies in multiple languages and from diverse sources.

## Conclusion

The findings of this study demonstrate that physical exercise can markedly enhance sleep quality in adolescents. A systematic exercise program lasting 3–12 weeks is beneficial for the sleep quality of adolescents, with the maximum benefit achieved at 12 weeks. Conducting the exercise at an appropriate frequency and ensuring each session lasts more than 30 min can also yield the most favorable results. Both aerobic and resistance exercise produced significant benefits, whereas combined exercise displayed no clear effect. These results provide a robust evidence base for refining exercise prescriptions and health management strategies, offering a practical and promising approach to ameliorating sleep problems caused by academic stress and laying a theoretical foundation for future research in this field. Educators, parents, and school leaders should thoughtfully organize a range of suitable physical activities that align with the physical and mental traits of adolescents. Moreover, they should actively motivate students to engage in various physical and mental exercises during their free time or weekends.

## Data Availability

The datasets presented in this study can be found in online repositories. The names of the repository/repositories and accession number(s) can be found in the article/Supplementary material.

## References

[1] Anastasiades PG De Vivo L Bellesi M Jones MW. Adolescent sleep and the foundations of prefrontal cortical development and dysfunction. Prog Neurobiol. (2022) 218:102338. doi: 10.1016/j.pneurobio.2022.102338, PMID: 35963360 PMC7616212

[2] Bacaro V Miletic K Crocetti E. A meta-analysis of longitudinal studies on the interplay between sleep, mental health, and positive well-being in adolescents. Int J Clin Health Psychol. (2024) 24:100424. doi: 10.1016/j.ijchp.2023.100424, PMID: 38125984 PMC10730350

[3] Ma H Li H Chen C Xu N Chen Q Ma J. Sleep effects on memory consolidation in adolescent English vocabulary memorization: investigating links between sleep quality, memory, and mood disturbance. Curr Psychol. (2024) 43:28429–37. doi: 10.1007/s12144-024-06384-9

[4] Matricciani L Olds T Petkov J. In search of lost sleep: secular trends in the sleep time of school-aged children and adolescents. Sleep Med Rev. (2012) 16:203–11. doi: 10.1016/j.smrv.2011.03.005, PMID: 21612957

[5] Husain S Morales KH Williamson AA Mayne SL Fiks AG Basner M . The neighborhood environment and sleep health in adolescents. Sleep Health. (2023) 9:512–8. doi: 10.1016/j.sleh.2023.05.010, PMID: 37391278 PMC10524795

[6] Bacaro V Andreose A Grimaldi M Natale V Tonetti L Crocetti E. The association between sleep patterns, educational identity, and school performance in adolescents. Brain Sci. (2023) 13:178. doi: 10.3390/brainsci13020178, PMID: 36831721 PMC9954600

[7] Buitron V Maron M Kudinova A Thompson E Barker DH Wolff JC. Sleep disturbance and suicidality in psychiatrically hospitalized adolescents: the role of specific emotion regulation domains. J Clin Psychol. (2023) 79:2515–28. doi: 10.1002/jclp.23558, PMID: 37329572 PMC10880543

[8] Zhang D Yang Y Zhai S Qu Y Li T Xie Y . Poor sleep pattern is associated with metabolic disorder during transition from adolescence to adulthood. Front Endocrinol. (2023) 14:1088135. doi: 10.3389/fendo.2023.1088135, PMID: 37033270 PMC10073678

[9] Wang Y Li B Zhang C Buxton OM Redline S Li X. Group-based sleep trajectories in children and adolescents: a systematic review. Sleep Med Rev. (2024) 75:101916. doi: 10.1016/j.smrv.2024.101916, PMID: 38461678

[10] Cen Y He J Zhong Y Zhou J Zeng J Huang G . The mediating role of sleep problems and depressed mood between psychological abuse/neglect and suicidal ideation in adolescent childhood: a multicentred, large sample survey in Western China. BMC Psychiatry. (2024) 24:64. doi: 10.1186/s12888-024-05503-x, PMID: 38262997 PMC10804755

[11] Viticchi G Di Stefano V Altamura C Falsetti L Torrente A Brunelli N . Effects of prophylactic drug therapies and anti-calcitonin peptide-related monoclonal antibodies on subjective sleep quality: an Italian multicenter study. Sleep Med. (2024) 117:87–94. doi: 10.1016/j.sleep.2024.03.026, PMID: 38518587

[12] Maiti R Mishra BR Jena M Mishra A Nath S. Effect of haloperidol and risperidone on serum melatonin and GAP-43 in patients with schizophrenia: a prospective cohort study. Clin Psychopharmacol Neurosci. (2021) 19:125–34. doi: 10.9758/cpn.2021.19.1.125, PMID: 33508796 PMC7851459

[13] Chen SY Khan F Talib S Toft S. Review of melatonin's effectiveness and the side effects on Alzheimer's disease. BJPsych Open. (2024) 10:S2–3. doi: 10.1192/bjo.2024.78

[14] Pillinger T Howes OD Correll CU Leucht S Huhn M Schneider-Thoma J . Antidepressant and antipsychotic side-effects and personalised prescribing: a systematic review and digital tool development. Lancet Psychiatry. (2023) 10:860–76. doi: 10.1016/S2215-0366(23)00262-6, PMID: 37774723 PMC10878984

[15] Vargas C Paoletti D De Stasio S Berenguer C. Sleep disturbances in autistic children and adolescents: a systematic review and meta-analysis of randomized controlled trials. Autism. (2025) 29:1661–73. doi: 10.1177/13623613251319391, PMID: 39968574

[16] Nobari H Banihashemi M Saedmocheshi S Prieto-González P Oliveira R. Overview of the impact of sleep monitoring on optimal performance, immune system function and injury risk reduction in athletes: a narrative review. Sci Prog. (2023) 106:00368504231206265. doi: 10.1177/00368504231206265, PMID: 37990537 PMC10666701

[17] Shah NM Bennett C Hassan H Kaltsakas G. Sleep disorders and exercise: a mini-review. J Thorac Dis. (2023) 15:5863–72. doi: 10.21037/jtd-23-17, PMID: 37969282 PMC10636486

[18] Post TE De Gioannis R Schmitz J Wittkowski M Schäper TM Wrobeln A . Resetting of the human circadian melatonin rhythm by ambient hypoxia. J Pineal Res. (2025) 77:e70029. doi: 10.1111/jpi.70029, PMID: 39821326 PMC11740168

[19] Tai D Falck RS Davis JC Vint Z Liu-Ambrose T. Can exercise training promote better sleep and reduced fatigue in people with chronic stroke? A systematic review. J Sleep Res. (2022) 31:e13675. doi: 10.1111/jsr.13675, PMID: 35762096

[20] Giannaki CD Sakkas GK Hadjigeorgiou GM Manconi M Bargiotas P. Unfolding the role of exercise in the management of sleep disorders. Eur J Appl Physiol. (2024) 124:2547–60. doi: 10.1007/s00421-024-05556-6, PMID: 39031176 PMC11365864

[21] Bello B Mohammed J Useh U. Effectiveness of physical activity programs in enhancing sleep outcomes among adolescents: a systematic review. Sleep Breathing. (2023) 27:431–9. doi: 10.1007/s11325-022-02675-2, PMID: 35771387

[22] Xu M Tian C Liang S Tong B Wu Y Zhou L . Comparative efficacy of exercise modalities on sleep quality in populations with sleep disorders: a systematic review and network meta-analysis. Sleep Med Rev. (2024) 73:101877. doi: 10.1016/j.smrv.2023.101877, PMID: 38006755

[23] Zhou X Kong Y Yu B Shi S He H. Effects of exercise on sleep quality in general population: meta-analysis and systematic review. Sleep Med. (2024) 10.1016/j.sleep.2024.10.036 39556996

[24] Page MJ McKenzie JE Bossuyt PM Boutron I Hoffmann TC Mulrow CD . The PRISMA 2020 statement: an updated guideline for reporting systematic reviews. BMJ. (2021) 74:790–9. doi: 10.1136/bmj.n71, PMID: 33781348 PMC8008539

[25] Chandler J Cumpston M Li T Page MJ Welch VJHW. Cochrane handbook for systematic reviews of interventions. Hoboken: Wiley (2019). 4 p. 10.1002/14651858.ED000142 PMC10284251 31643080

[26] Higgins JP. Cochrane handbook for systematic reviews of interventions Cochrane Collaboration and John Wiley & Sons Ltd (2008).

[27] Cumpston M Li T Page MJ Chandler J Welch VA Higgins JP . Updated guidance for trusted systematic reviews: a new edition of the Cochrane handbook for systematic reviews of interventions. Cochrane Database Syst Rev. (2019) 2019:ED000142 10.1002/14651858.ED000142 PMC10284251 31643080

[28] Borenstein M Hedges LV Higgins JP Rothstein HR. A basic introduction to fixed-effect and random-effects models for meta-analysis. Res Synth Methods. (2010) 1:97–111. doi: 10.1002/jrsm.12, PMID: 26061376

[29] Viechtbauer W Cheung MWL. Outlier and influence diagnostics for meta-analysis. Res Synth Methods. (2010) 1:112–25. doi: 10.1002/jrsm.11, PMID: 26061377

[30] Kalak N Gerber M Kirov R Mikoteit T Yordanova J Pühse U . Daily morning running for 3 weeks improved sleep and psychological functioning in healthy adolescents compared with controls. J Adolesc Health. (2012) 51:615–22. doi: 10.1016/j.jadohealth.2012.02.020, PMID: 23174473

[31] Baldursdottir B Taehtinen RE Sigfusdottir ID Krettek A Valdimarsdottir HB. Impact of a physical activity intervention on adolescents’ subjective sleep quality: a pilot study. Glob Health Promot. (2017) 24:14–22. doi: 10.1177/1757975915626112, PMID: 27173502

[32] Santiago LC Lyra MJ Germano-Soares AH Lins-Filho OL Queiroz DR Prazeres TM . Effects of strength training on sleep parameters of adolescents: a randomized controlled trial. J Strength Cond Res. (2022) 36:1222–7. doi: 10.1519/JSC.0000000000003629, PMID: 32379244

[33] Hu Y Duan X Zhang Z Lu C Zhang Y. Effects of adverse events and 12-week group step aerobics on sleep quality in Chinese adolescents. Children. (2023) 10:1253. doi: 10.3390/children10071253, PMID: 37508750 PMC10377765

[34] Parveen A Kalra S Awasthi S Ajmera P Rai RH Miraj M . Effect of mat Pilates intervention on sleep quality in adolescent girls: a single blinded randomised controlled trial. Clin Epidemiol Global Health. (2024) 30:101791. doi: 10.1016/j.cegh.2024.101791

[35] Wang K Li Y Liu S Liu H Zhang T Luo J. Can an intervention integrating sports and medicine improve children’s health more effectively? Monitoring based on sleep, body mass index, and heart rate variability. J Glob Health. (2024) 14:04040. doi: 10.7189/jogh.14.04040, PMID: 38635801 PMC11026036

[36] da Silva JM Castilho dos Santos G de Oliveira Barbosa R de Souza Silva TM Correa RC da Costa BGG . Effects of a school-based physical activity intervention on mental health indicators in a sample of Brazilian adolescents: a cluster randomized controlled trial. BMC Public Health. (2025) 25:539. doi: 10.1186/s12889-025-21620-y, PMID: 39930438 PMC11809091

[37] Kao HF Lin CF Lin IT Hung YC Chang TW Ho CC. Improved sleep quality and sleep duration after an 8-week exergaming intervention for exercise training among elementary schoolchildren in Taiwan. Children. (2025) 12:180. doi: 10.3390/children12020180, PMID: 40003282 PMC11854434

[38] Steinberg LD Lerner RM. Handbook of adolescent psychology. New York: John Wiley & Sons (2004).

[39] Rothstein H. R. Sutton A. J. Borenstein M. (Eds.) (2005). Publication bias in meta-analysis. Publication bias in meta-analysis: prevention, assessment and adjustments (Wiley Online Library), 1–7.

[40] He D Zhang C Li R Zhang X. Baduanjin exercise for negative emotion of patients undergoing chemotherapy: a systematic review and meta-analysis. Support Care Cancer. (2024) 32:608. doi: 10.1007/s00520-024-08804-9, PMID: 39172232

[41] Athanasiou N Bogdanis GC Mastorakos G. Endocrine responses of the stress system to different types of exercise. Rev Endocr Metabol Disorders. (2023) 24:251–66. doi: 10.1007/s11154-022-09758-1, PMID: 36242699 PMC10023776

[42] Pahlavani HA. Possible role of exercise therapy on depression: effector neurotransmitters as key players. Behav Brain Res. (2024) 459:114791. doi: 10.1016/j.bbr.2023.114791, PMID: 38048912

[43] Cakin Memik N Hunc F Kalayci S Demir N Senturk E Yildiz Gundogdu O . Assessment of plasma-endogenous opioid neuropeptide levels and psychometric properties of non-suicidal self-injury in adolescents. Arch Suicide Res. (2023) 27:749–68. doi: 10.1080/13811118.2022.2066494, PMID: 35499526

[44] Yang Q Xie R Wang D Li J Zhang R Li W . How to survive the long night? Longitudinal relationship between sleep problems and suicidal behavior among adolescents: the serial mediating roles of negative emotion, self-control, and nonsuicidal self-injury. Suicide Life Threat Behav. (2024) 54:349–60. doi: 10.1111/sltb.13046, PMID: 38284480

[45] Li L Wang C Wang D Li H Zhang S He Y . Optimal exercise dose and type for improving sleep quality: a systematic review and network meta-analysis of RCTs. Front Psychol. (2024) 15:1466277. doi: 10.3389/fpsyg.2024.1466277, PMID: 39421847 PMC11484100

[46] Furuya S Oku T Nishioka H Hirano M. Surmounting the ceiling effect of motor expertise by novel sensory experience with a hand exoskeleton. Sci Robot. (2025) 10:eadn3802. doi: 10.1126/scirobotics.adn3802, PMID: 39813311

[47] de la Torre-Cruz MJ Rusillo-Magdaleno A Solas-Martínez JL Moral García JE. Physical activity and subjective vitality in female university students: the mediating role of decisional balance and enjoyment of the activity. Behav Sci. (2024) 14:685. doi: 10.3390/bs14080685, PMID: 39199081 PMC11352169

[48] Tyler J Podaras M Richardson B Roeder N Hammond N Hamilton J . High intensity interval training exercise increases dopamine D2 levels and modulates brain dopamine signaling. Front Public Health. (2023) 11:1257629. doi: 10.3389/fpubh.2023.1257629, PMID: 38192549 PMC10773799

[49] Hou LJ Geng YX Ke LI Huang ZY Mao LQ. Research on the role of dopamine in regulating sleep and wakefulness through exercise. Prog Biochem Biophys. (2025) 52:88–98.

[50] Fernández-Cuevas I Torres G Sillero-Quintana M Navandar A. Thermographic assessment of skin response to strength training in young participants. J Therm Anal Calorim. (2023) 148:3407–15. doi: 10.1007/s10973-023-11978-9

[51] Haghayegh S Khoshnevis S Smolensky MH Hermida RC Castriotta RJ Schernhammer E . Novel temperature-controlled sleep system to improve sleep: a p roof-of-concept study. J Sleep Res. (2022) 31:e13662. doi: 10.1111/jsr.13662, PMID: 35852479

[52] Fan Y Wang Y Gu P Han J Tian Y. How temperature influences sleep. Int J Mol Sci. (2022) 23:12191. doi: 10.3390/ijms232012191, PMID: 36293048 PMC9603733

[53] Li M Wang Q Shen J. The impact of physical activity on mental health during COVID-19 pandemic in China: a systematic review. Int J Environ Res Public Health. (2022) 19:6584. doi: 10.3390/ijerph19116584, PMID: 35682172 PMC9180501

[54] Whitworth JW Nosrat S SantaBarbara NJ Ciccolo JT. High intensity resistance training improves sleep quality and anxiety in individuals who screen positive for posttraumatic stress disorder: a randomized controlled feasibility trial. Ment Health Phys Act. (2019) 16:43–9. doi: 10.1016/j.mhpa.2019.04.001

[55] Hu YX Liu XM Zhang NX Ma ZY Zhu Z Cao ZB. The effects of resistance are superior to aerobic exercise in improving delayed sleep-wake phase disorder in male college students. Sleep Med. (2025) 128:29–36. doi: 10.1016/j.sleep.2025.01.029, PMID: 39879677

[56] Tse AC Lee PH Zhang J Chan RC Ho AW Lai EW. Effects of exercise on sleep, melatonin level, and behavioral functioning in children with autism. Autism. (2022) 26:1712–22. doi: 10.1177/13623613211062952, PMID: 35083939

[57] Estaphan S Wadley GD Todd G Towstoless M Hryciw DH Lexis L . Unpacking and validating the “physiological adaptation” core concept of physiology. Adv Physiol Educ. (2023) 47:831–7. doi: 10.1152/advan.00083.2023, PMID: 37650145

[58] Weakley J Schoenfeld BJ Ljungberg J Halson SL Phillips SM. Physiological responses and adaptations to lower load resistance training: implications for health and performance. Sports Med Open. (2023) 9:28. doi: 10.1186/s40798-023-00578-4, PMID: 37171517 PMC10182225

[59] Tworoger SS Yasui Y Vitiello MV Schwartz RS Ulrich CM Aiello EJ . Effects of a yearlong moderate-intensity exercise and a stretching intervention on sleep quality in postmenopausal women. Sleep. (2003) 26:830–6. doi: 10.1093/sleep/26.7.830, PMID: 14655916

[60] Leota J Presby DM Le F Czeisler MÉ Mascaro L Capodilupo ER . Dose-response relationship between evening exercise and sleep. Nat Commun. (2025) 16:3297. doi: 10.1038/s41467-025-58271-x, PMID: 40234380 PMC12000559

[61] Brellenthin AG Lee DC. Comparative effects of aerobic, resistance, and combined exercise on sleep. Circulation. (2022) 145:A038–8.

[62] Wang ZY Deng YL Zhou TY Jiang ZY Liu Y Liu BF . The effects of exercise interventions on depressive symptoms in stroke patients: a systematic review and meta-analysis. Front Physiol. (2025) 16:1492221. doi: 10.3389/fphys.2025.1492221, PMID: 40166715 PMC11955706

[63] Li K Omar Dev RD Li W. Physical activity and happiness of college students: chain mediating role of exercise attitude and sleep quality. Front Public Health. (2025) 13:1544194. doi: 10.3389/fpubh.2025.1544194, PMID: 39911221 PMC11794275

[64] Zhao JL Jiang WT Wang X Cai ZD Liu ZH Liu GR. Exercise, brain plasticity, and depression. CNS Neurosci Ther. (2020) 26:885–95. doi: 10.1111/cns.13385, PMID: 32491278 PMC7415205

[65] Li MY Huang MM Li SZ Tao J Zheng GH Chen LD. The effects of aerobic exercise on the structure and function of DMN-related brain regions: a systematic review. Int J Neurosci. (2017) 127:634–49. doi: 10.1080/00207454.2016.1212855, PMID: 27412353

[66] Moir ME Corkery AT Miller KB Pearson AG Loggie NA Apfelbeck AA . The independent and combined effects of aerobic exercise intensity and dose differentially increase post-exercise cerebral shear stress and blood flow. Exp Physiol. (2024) 109:1796–805. doi: 10.1113/EP091856, PMID: 39141846 PMC11442852

[67] Assis GGD Gasanov EV De Sousa MBC Kozacz A Murawska-Cialowicz E. Brain-derived neurotrophic factor, a link of aerobic metabolism to neuroplasticity. J Physiol Pharmacol. (2018) 69:351–8. 10.26402/jpp.2018.3.12 30342429

[68] Redolfi S Bettinzoli M Venturoli N Ravanelli M Pedroni L Taranto-Montemurro L . Attenuation of obstructive sleep apnea and overnight rostral fluid shift by physical activity. Am J Respir Crit Care Med. (2015) 191:856–8. doi: 10.1164/rccm.201412-2192LE, PMID: 25830523

[69] Kasai T Motwani SS Yumino D Mak S Newton GE Bradley TD. Differing relationship of nocturnal fluid shifts to sleep apnea in men and women with heart failure. Circ Heart Fail. (2012) 5:467–74. doi: 10.1161/CIRCHEARTFAILURE.111.965814, PMID: 22679060

[70] Tang R Pan J Huang Y Ren X. Efficacy comparison of aerobic exercise, combined exercise, oropharyngeal exercise and respiratory muscle training for obstructive sleep apnea: a systematic review and network meta-analysis. Sleep Med. (2024) 124:582–90. doi: 10.1016/j.sleep.2024.10.026, PMID: 39476608

[71] Vasey C McBride J Penta K. Circadian rhythm dysregulation and restoration: the role of melatonin. Nutrients. (2021) 13:3480. doi: 10.3390/nu13103480, PMID: 34684482 PMC8538349

[72] Niu J Li Y Zhou Q Liu X Yu P Gao F . The association between physical activity and delayed neurocognitive recovery in elderly patients: a mediation analysis of pro-inflammatory cytokines. Aging Clin Exp Res. (2024) 36:192. doi: 10.1007/s40520-024-02846-z, PMID: 39259352 PMC11390811

[73] Ayari S Abellard A Carayol M Guedj E Gavarry O. A systematic review of exercise modalities that reduce pro-inflammatory cytokines in humans and animals' models with mild cognitive impairment or dementia. Exp Gerontol. (2023) 175:112141. doi: 10.1016/j.exger.2023.112141, PMID: 36898593

[74] Pedersen BK. Exercise-induced myokines and their role in chronic diseases. Brain Behav Immun. (2011) 25:811–6. doi: 10.1016/j.bbi.2011.02.010, PMID: 21354469

[75] Li W Fang W Zhang Y Chen Q Shentu W Lai Q . Research progress on resistance exercise therapy for improving cognitive function in patients with AD and muscle atrophy. Front Aging Neurosci. (2025) 17:1552905. doi: 10.3389/fnagi.2025.1552905, PMID: 40271180 PMC12016217

[76] Rodrigues NR Macedo GE Martins IK de Brum Vieira P Kich KG Posser T . Sleep disturbance induces a modulation of clock gene expression and alters metabolism regulation in drosophila. Physiol Behav. (2023) 271:114334 37595818 10.1016/j.physbeh.2023.114334

[77] Heo SJ Jee YS. Intensity-effects of strengthening exercise on thigh muscle volume, pro-or anti-inflammatory cytokines, and immunocytes in the older adults: a randomized controlled trial. Arch Gerontol Geriatr. (2024) 116:105136. doi: 10.1016/j.archger.2023.105136, PMID: 37541052

[78] Li TS Wang R Su X Wang XQ. Effect and mechanisms of exercise for complex regional pain syndrome. Front Mol Neurosci. (2023) 16:1167166. doi: 10.3389/fnmol.2023.1167166, PMID: 37206984 PMC10188984

