# Supplementary material for: Effects of exercise interventions on sleep quality in adolescents: a systematic review and meta-analysis
Source: Front Public Health. 2025 Sep 3;13:1623506. doi: 10.3389/fpubh.2025.1623506 (PMC12452866; doi:10.3389/fpubh.2025.1623506)
Supplement: Supplementary file 1 [file Data_Sheet_1.ZIP › Literature Search Record/Retrieval keywords.docx]

PUBMED

("Exercise"[MeSH Terms] OR ("Exercise"[Title/Abstract] OR "physical activity"[Title/Abstract] OR "physical training"[Title/Abstract] OR "aerobic exercise"[Title/Abstract] OR "physical exercise"[Title/Abstract] OR "baduanjin"[Title/Abstract] OR "qigong"[Title/Abstract] OR "tai chi"[Title/Abstract] OR "yoga"[Title/Abstract] OR "pilate*"[Title/Abstract] OR "resistance training"[Title/Abstract] OR "physical training"[Title/Abstract] OR "strength training"[Title/Abstract] OR "walk"[Title/Abstract] OR "swim"[Title/Abstract] OR "fitness"[Title/Abstract])) AND ("Sleep"[MeSH Terms] OR ("Sleep"[Title/Abstract] OR "sleep quality"[Title/Abstract] OR "sleep disturbance*"[Title/Abstract] OR "sleep maintenance"[Title/Abstract] OR "sleep disorder*"[Title/Abstract] OR "sleep problem*"[Title/Abstract] OR "insomnia"[Title/Abstract] OR "sleeplessness"[Title/Abstract] OR "sleep duration*"[Title/Abstract] OR "sleep health"[Title/Abstract])) AND ("Adolescent"[MeSH Terms] OR ("adolescent*"[Title/Abstract] OR "youth*"[Title/Abstract] OR "teen*"[Title/Abstract] OR "school student*"[Title/Abstract])) AND ("randomized controlled trial"[Title/Abstract] OR "randomized"[Title/Abstract] OR "placebo"[Title/Abstract])

Cochrane

Embase

('exercise'/exp OR 'exercise':ab,ti OR 'physical activity':ab,ti OR 'aerobic exercise':ab,ti OR 'physical exercise':ab,ti OR 'baduanjin':ab,ti OR 'qigong':ab,ti OR 'tai chi':ab,ti OR 'yoga':ab,ti OR 'pilate*':ab,ti OR 'resistance training':ab,ti OR 'physical training':ab,ti OR 'strength training':ab,ti OR 'walk':ab,ti OR 'swim':ab,ti OR 'fitness':ab,ti) AND ('sleep'/exp OR 'sleep':ab,ti OR 'sleep quality':ab,ti OR 'sleep disturbance*':ab,ti OR 'sleep maintenance':ab,ti OR 'sleep disorder*':ab,ti OR 'sleep problem*':ab,ti OR 'insomnia':ab,ti OR 'sleeplessness':ab,ti OR 'sleep duration*':ab,ti OR 'sleep health':ab,ti) AND ('adolescent'/exp OR 'adolescent*':ab,ti OR 'youth*':ab,ti OR 'teen*':ab,ti OR 'school student*':ab,ti) AND ('randomized controlled trial':ab,ti OR 'randomized':ab,ti OR 'placebo':ab,ti)

WEB OF SCIENCE

(((((((((((((((TS=(Exercise)) OR TS=(physical activity)) OR TS=(physical training)) OR TS=(aerobic exercise)) OR TS=(physical exercise)) OR TS=(baduanjin)) OR TS=(qigong)) OR TS=(tai chi)) OR TS=(yoga)) OR TS=(pilate*)) OR TS=(resistance training)) OR TS=(physical training)) OR TS=(strength training)) OR TS=(walk)) OR TS=(swim)) OR TS=(fitness)
