# Supplementary figures and images for: Effects of exercise interventions on sleep quality in adolescents: a systematic review and meta-analysis
Source: Front Public Health. 2025 Sep 3;13:1623506. doi: 10.3389/fpubh.2025.1623506 (PMC12452866; doi:10.3389/fpubh.2025.1623506)

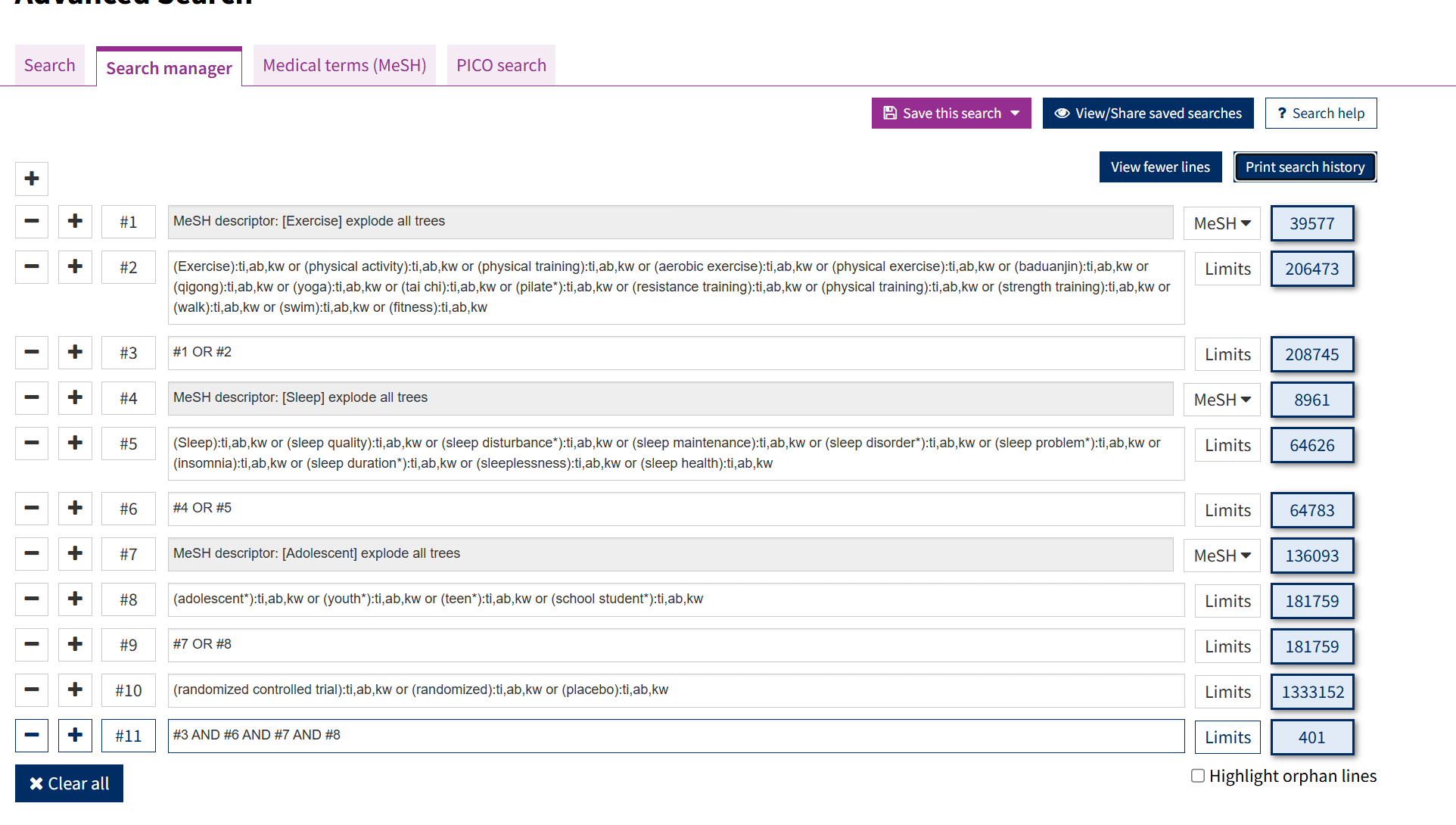

Supplement: Supplementary file 1 [file Data_Sheet_1.ZIP › Literature Search Record/Screenshot of search records/1744808261923.jpg]

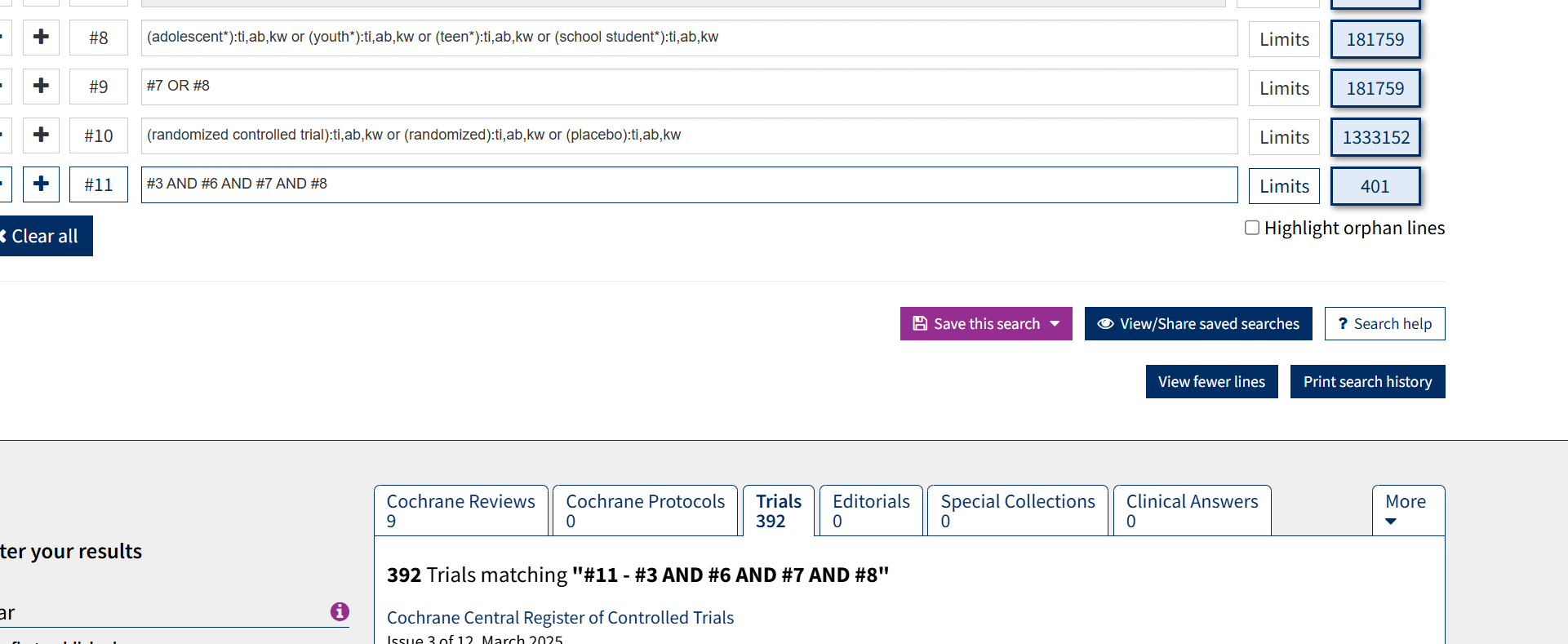

Supplement: Supplementary file 1 [file Data_Sheet_1.ZIP › Literature Search Record/Screenshot of search records/1744808280910.jpg]

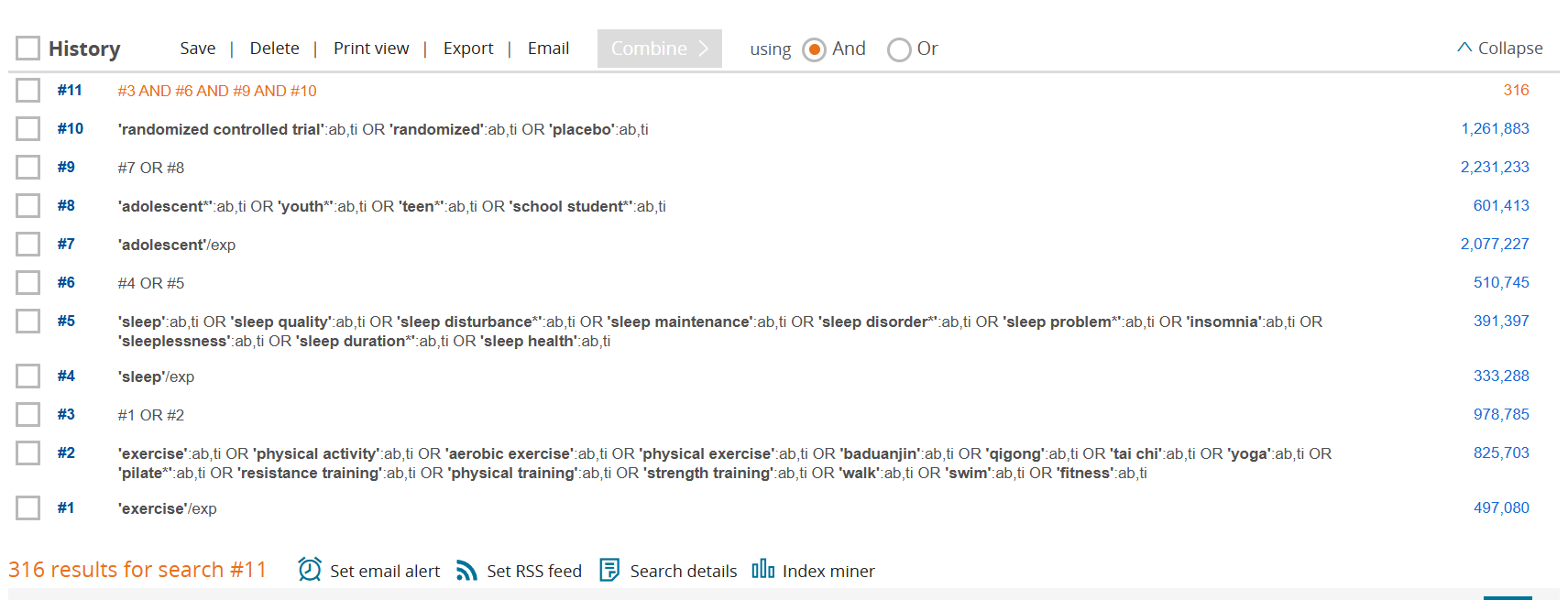

Supplement: Supplementary file 1 [file Data_Sheet_1.ZIP › Literature Search Record/Screenshot of search records/1744812052676.jpg]

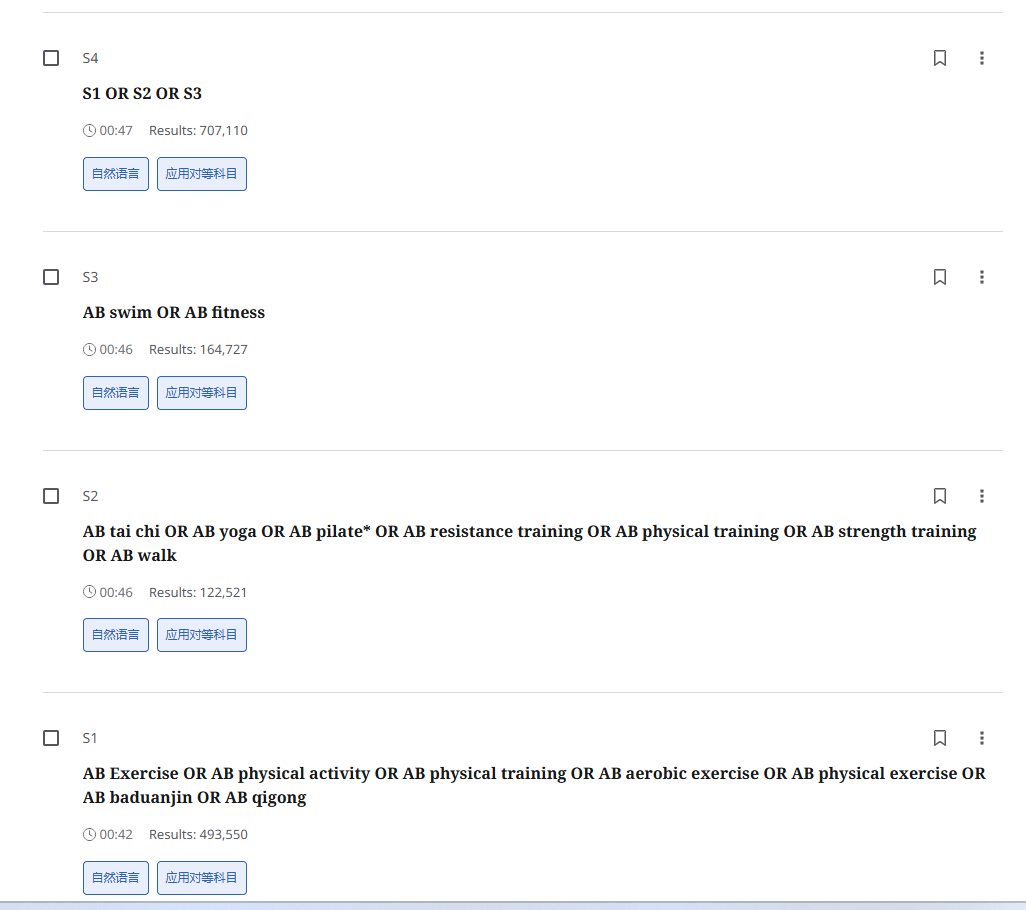

Supplement: Supplementary file 1 [file Data_Sheet_1.ZIP › Literature Search Record/Screenshot of search records/1744824307540.jpg]

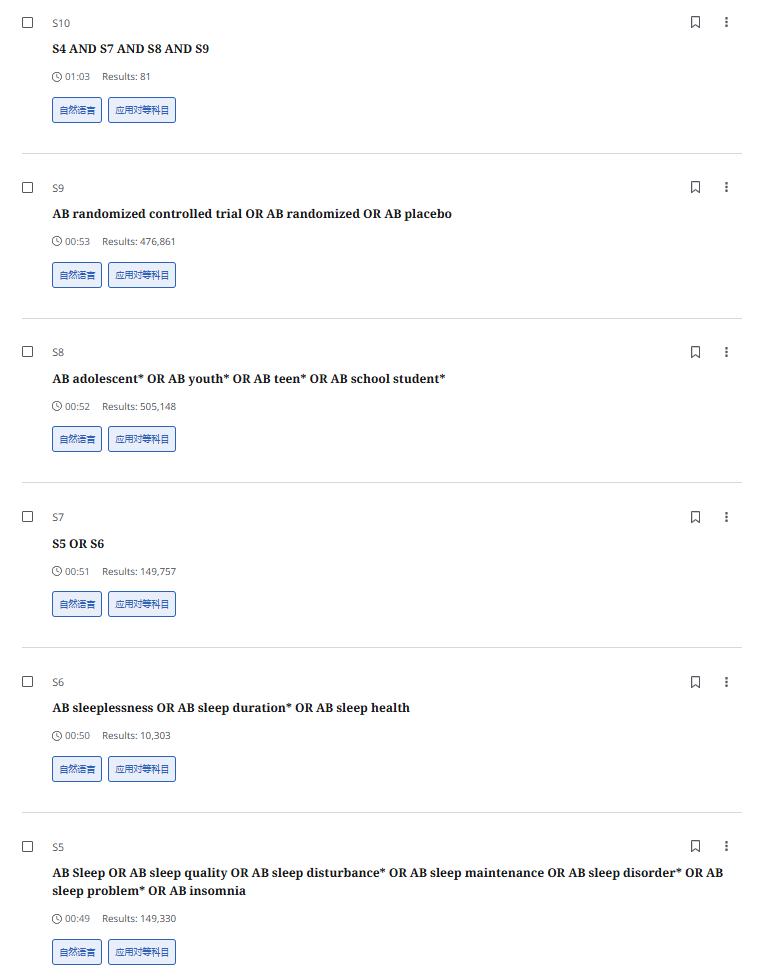

Supplement: Supplementary file 1 [file Data_Sheet_1.ZIP › Literature Search Record/Screenshot of search records/EBSCO 2.jpg]

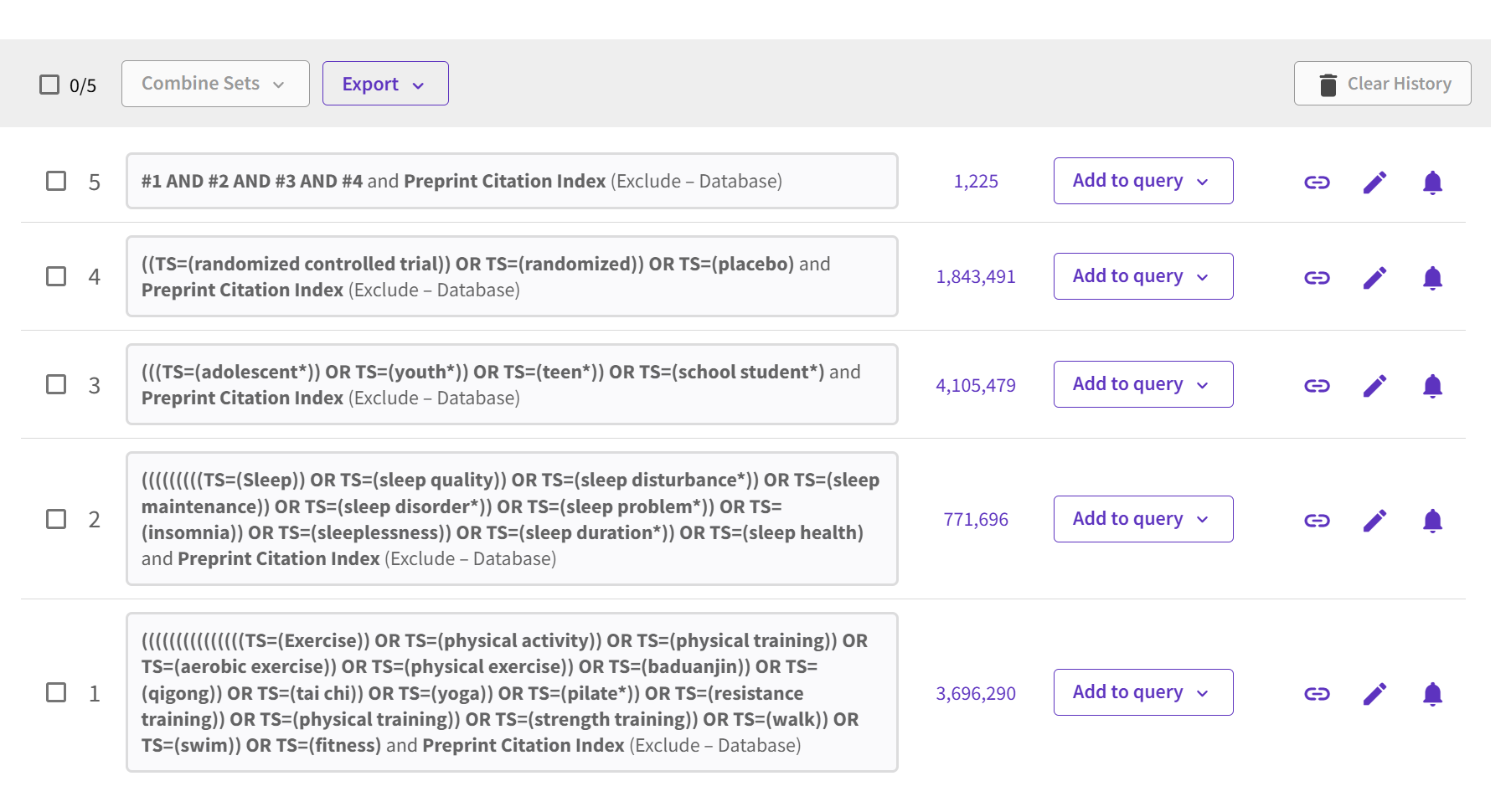

Supplement: Supplementary file 1 [file Data_Sheet_1.ZIP › Literature Search Record/Screenshot of search records/ebsco.jpg]
